# Mass spectrometric profiling of DNA adducts in the human stomach associated with damage from environmental factors

**DOI:** 10.1186/s41021-021-00186-2

**Published:** 2021-04-09

**Authors:** Ippei Ohnishi, Yuji Iwashita, Yuto Matsushita, Shunsuke Ohtsuka, Takashi Yamashita, Keisuke Inaba, Atsuko Fukazawa, Hideto Ochiai, Keigo Matsumoto, Nobuhito Kurono, Yoshitaka Matsushima, Hiroki Mori, Shioto Suzuki, Shohachi Suzuki, Fumihiko Tanioka, Haruhiko Sugimura

**Affiliations:** 1 Department of Tumor Pathology, Hamamatsu University School of Medicine, 1-20-1 Handayama, Higashi-ku, Hamamatsu, Shizuoka, 431-3192 Japan; 2 Pathology Division, Iwata City Hospital, 512-3 Ohkubo, Iwata, Shizuoka, 438-8550 Japan; 3 Department of Urology, Hamamatsu University School of Medicine, 1-20-1 Handayama, Higashi-ku, Hamamatsu, Shizuoka, 431-3192 Japan; 4 Hamamatsu Medical Center, 328 Tomitsuka-cho, Naka-ku, Hamamatsu, Shizuoka, 432-8580 Japan; 5 First Department of Surgery, Hamamatsu University School of Medicine, 1-20-1 Handayama, Higashi-ku, Hamamatsu, Shizuoka, 431-3192 Japan; 6 Surgery Division, Iwata City Hospital, 512-3 Ohkubo, Iwata, Shizuoka, 438-8550 Japan; 7 Department of Chemistry, Hamamatsu University School of Medicine, 1-20-1 Handayama, Higashi-ku, Hamamatsu, Shizuoka, 431-3192 Japan; 8 Department of Agricultural Chemistry, Tokyo University of Agriculture, 1-1-1 Sakuragaoka, Setagaya-ku, Tokyo, 156-8502 Japan

**Keywords:** DNA adduct, DNA adductome, DNA adductomics, Mutagen, Exposure, Stomach, Gastric cancer, Liquid chromatography/tandem mass spectrometry

## Abstract

**Background:**

A comprehensive understanding of DNA adducts, one of the most plausible origins of cancer mutations, is still elusive, especially in human tissues in clinical settings. Recent technological developments have facilitated the identification of multiple DNA adducts in a single experiment. Only a few attempts toward this “DNA adductome approach” in human tissues have been reported. Geospatial information on DNA adducts in human organs has been scarce.

**Aim:**

Mass spectrometry of human gastric mucosal DNA was performed to identify DNA adducts associated with environmental factors.

**Materials and methods:**

From 59 subjects who had received gastrectomy for gastric cancer, 306 samples of nontumor tissues and 15 samples of tumors (14 cases) were taken for DNA adductome analysis. Gastric nontumor tissue from autopsies of 7 subjects without gastric cancer (urothelial cancer, hepatocellular carcinoma, lung cancer each; the other four cases were without any cancers) was also investigated. Briefly, DNA was extracted from each sample with antioxidants, digested into nucleosides, separated by liquid chromatography, and then electrospray-ionized. Specific DNA adducts were identified by mass/charge number and column retention time compared to standards. Information on lifestyle factors such as tobacco smoking and alcohol drinking was taken from the clinical records of each subject.

**Results:**

Seven DNA adducts, including modified bases, C5-methyl-2′-deoxycytidine, 2′-deoxyinosine, C5-hydroxymethyl-2′-deoxycytidine, N6-methyl-2′-deoxyadenosine, 1,N6-etheno-2′-deoxyadenosine, N6-hydroxymethyl-2′-deoxyadenosine, and C8-oxo-2′-deoxyguanosine, were identified in the human stomach and characterized. Intraindividual differences according to the multiple sites of these adducts were noted but were less substantial than interindividual differences. N6-hydroxymethyl-2′-deoxyadenosine was identified in the human stomach for the first time. The amount of C5-hydroxymethyl-2′-deoxycytidine was higher in the stomachs of subjects without gastric cancer than in the nontumor and tumor portions of the stomach in gastric cancer patients. Higher levels of 1,N6-etheno-2′-deoxyadenosine were detected in the subjects who reported both smoking and drinking than in those without these habits. These DNA adducts showed considerable correlations with each other.

**Conclusions:**

We characterized 7 DNA adducts in the nontumor portion of the human stomach in both gastric cancer subjects and nongastric cancer subjects. A reduction in C5-hydroxymethyl-dC even in the nontumor mucosa of patients with gastric cancer was observed. Smoking and drinking habits significantly influenced the quantity of one of the lipid peroxidation-derived adducts, etheno-dA. A more expansive DNA adductome profile would provide a comprehensive understanding of the origin of human cancer in the future.

**Supplementary Information:**

The online version contains supplementary material available at 10.1186/s41021-021-00186-2.

## Introduction

The human body is influenced by various environmental factors, which occasionally may be harmful or carcinogenic [1–5]. Environmental factors encompass a broad range of chemical carcinogenic substances, inflammatory conditions caused by exogenous and endogenous infection, irradiation (including ultraviolet light from the sun) and lifestyles related to the factors listed above, such as smoking, alcohol consumption, diet and nutrition. Aging itself also has a toxic effect on the human body. It is, however, very difficult to precisely clarify the individual effects of these numerous influences in the human body through experimental procedures [4]. In human tissue harboring cancers, surrounding nontumor cells have various internal and external factors that may have played a role in the cancer mutagenesis. Mutations and genomic amplifications have been reported in some precancerous sites of the stomach [6, 7]. In addition, promoter methylation in gastric and urothelial mucosa has been widely investigated [8, 9]. Reports of mutations found in “normal” tissues by using deep sequencing have given us new insight into our understanding of the origin of human cancer [10, 11]. The initial events responsible for these carcinogenic and irreversible mutations involve DNA adduct formation, which is the focus of this paper. Due to internal or external factors, chemically reactive substances bind to DNA and form DNA adducts. Certain DNA adducts may impede accurate DNA replication and generate mispairing which contribute to DNA mutations [12]. The demonstration of the existence of DNA adducts, especially in human tissue, continues to be a challenge to researchers, due in part to limitations of the available methodologies. In 2003, Abdul-Momen et al. [13] reported the identification of 5 different spots on a ^32^P-postlabeling autoradiogram of 19 human adult stomach mucosae as unique DNA adducts, but this methodology did not allow them to analyze the type of DNA adducts. DNA adductomics, the recently developed concept of comprehensive detection of numerous DNA adducts in a single experiment, usually by using liquid chromatography and tandem mass spectrometry (LC-MS/MS), has facilitated unambiguous DNA adduct detection [14–16]. Theoretically, we can obtain comprehensive information on numerous DNA adduct profiles in one measurement, provided that appropriate standards are available [17]. In this report, we present DNA adduct detection from several loci from the mucosa of individual stomachs resected for gastric cancer or autopsied for other reasons. Comprehensive identification of DNA adducts as a form of DNA damage associated with environmental factors in the human gastric mucosa would reveal the pivotal steps in human gastric carcinogenesis. This adductomics approach will clarify various molecular events that occur in an ordinary living environment and will provide specific tools to detect and prevent DNA damage that may lead to carcinogenesis.

## Materials and methods

### Subjects

**Fig. 1:**
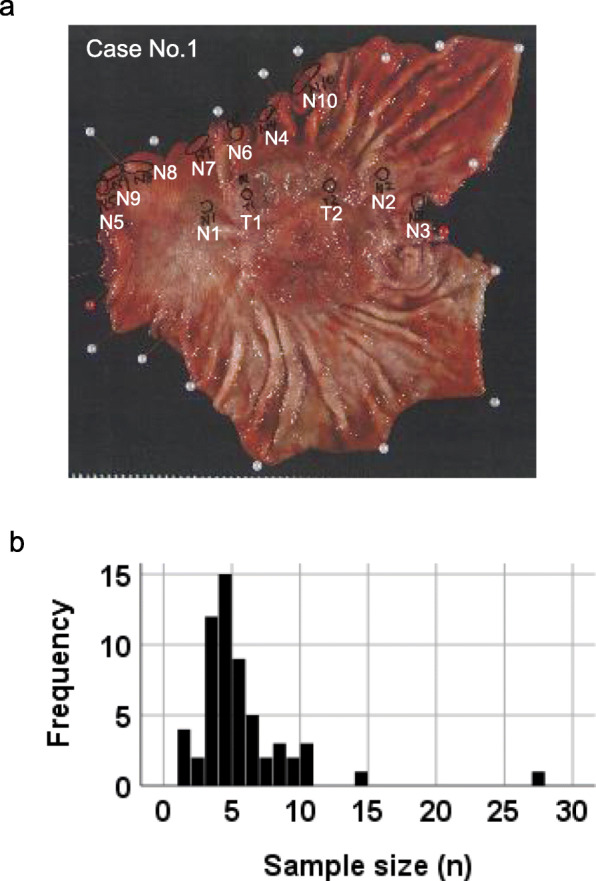
Human stomach from which mucosal tissue samples were taken from multiple sites. (a) Example of macroscopic view after multisite sampling in the stomach, resected for gastric cancer in this case. Mucosal tissues were taken: 10 from the nontumor area and 2 from the tumor area (N1-N10, T1-T2). (b) A histogram of the numbers of sampling sites of nontumor areas in gastric cancer cases. The x-axis represents the sample size (numbers taken for DNA analysis) taken from an individual and the y-axis represents the number of individuals with that sample size. Among the 59 cases in this study, the maximum number of samples collected from one case was 27

Collection of samples was conducted based on the Japanese Society of Pathology Guidelines on the handling of pathological tissue samples for genomic research [18]. From the residual pathological tissue from 59 cases of stomach resected for gastric cancer, several mucosal portions of up to 1 cm^2^, which were verified macroscopically as tumorous or nontumorous regions (no contamination with tumor cells or muscle layers) by attending pathologists, were snap-frozen in liquid nitrogen and stored at − 80 °C until use for DNA isolation. A representative case with multiple samples is shown in Fig. 1a. In 9 cases of total gastrectomy, mucosal tissues were taken from the upper, middle, and lower third parts of the stomach. In 42 cases of subtotal gastrectomy (distal gastrectomy), mucosal tissues were taken from the lower and middle parts of the stomach. In 4 cases of cardiac gastrectomy, mucosal tissues were taken from the upper and middle zones of the stomach. In the other 4 cases, mucosal tissues were taken from only one zone. The zones for sample retrieval, upper (U), middle (M), and lower (L), were determined according to the Japanese Classification System (JCS) by the Japanese Gastric Cancer Association (the upper portion corresponds approximately to the fundus and cardia, the middle to the transitional zone and the body, and the lower third to the prepyloric region and antrum of the stomach). Two samples of gastroesophageal junctions were categorized as U. The cancer and surrounding tissue from each case were fixed, embedded, and stained for microscopic evaluation. Histological classification of gastric cancer was based on the Japanese Classification of Gastric Carcinoma. In 14 cases, corresponding tumor tissues were also collected as frozen samples. Twenty samples of mucosal tissues were also taken from the three zones (U, M and L) of the stomachs of 7 autopsy patients who did not have gastric cancer, and those tissues were also frozen in liquid nitrogen. All procedures were performed according to the Declaration of Helsinki, and the use of residual pathological tissue in this research was approved by the Institutional Review Board of Hamamatsu University School of Medicine (IRB 20–011) and Iwata City Hospital.

### Lifestyle information

Lifestyle information was taken from the clinical and nursing records. For smoking, we classified patients into never smokers, current smokers, and former smokers. Estimation of alcohol equivalents was made with reference to the past literature [19]. We collected the frequency and amounts of various alcohol beverages consumed by each individual according to self-reported records, and the total intake was calculated from ethanol equivalents (grams) per drink. According to previous papers, we assumed that 1 cup of Japanese sake, shochu and whisky contained 24 g, 24 g and 20 g of ethanol, respectively. In the case of beer, the equation “amount (ml) x 0.05 x 0.8 = the content of ethanol (gram)” was used [20–23].

### DNA purification

Mucosal tissues were quickly frozen in liquid nitrogen and kept in a deep freezer at − 80 °C until DNA purification. Genomic DNA was purified from samples of human gastric tissue using a Gentra Puregene Tissue Kit™ (QIAGEN, Venlo, Netherlands). Approximately 100 mg of frozen tissue sample was ground in liquid nitrogen with a mortar and pestle and mixed with 3 ml Cell Lysis Solution™. The samples were treated with 15 μl Proteinase K at 55 °C for 3 h or overnight until tissues were completely lysed and were also treated with 15 μl of RNase A at 37 °C for 45 min. After incubation for 3 min on ice to quickly cool, 1 ml Protein Precipitation Solution™ was added to the mixture, which was then vortexed vigorously for 20 s at high speed. After centrifugation for 10 min at 2000 *g*, 3 ml isopropanol was added to the supernatant from the previous step and then centrifuged at 2000 *g* for 3 min to precipitate the genomic DNA. Precipitates were washed with 70% ethanol and dried. The dried genomic DNA was rehydrated with 50 μl distilled water containing 1 mM deferoxamine, and the DNA concentration was adjusted to 667 ng/μl.

### Enzymatic hydrolysis of DNA

The enzymatic hydrolysis step was conducted using the protocol provided with the 8-OHdG Assay Preparation Reagent Set (WAKO, Osaka, Japan). Briefly, 45 μl of DNA solution was heated at 98 °C for 2 min and chilled on ice for 10 min. Then, 5.7 μl of Acetic Acid Buffer™ and 3.0 μl of Nuclease P1 Solution™ were added to the DNA solution. After incubation at 37 °C for 30 min, 6.0 μl Tris Buffer and 0.3 μl Alkaline Phosphatase Solution™ were added to the mixture. After incubation at 37 °C for 30 min, the enzymes were removed using Nanosep™ Centrifugal Devices 3 K (Pall Corporation, Port Washington, NY, USA) by centrifugation at 14,000 *g* at 4 °C for 20 min. Digested samples were stored at − 30 °C until use for LC-MS/MS analysis.

### Standard compounds for DNA adducts

Commercially available chemical compounds were purchased from several vendors: (2′-deoxycytidine (dC), 2′-deoxythymidine (dT), 2′-deoxyguanosine (dG), 2′-deoxyadenosine (dA), 1,N6-etheno-2′-deoxyadenosine (etheno-dA), and C8-oxo-2′-deoxyguanosine (C8-oxo-dG) from Sigma-Aldrich Japan (Sigma-Aldrich, Tokyo, Japan); C5-methyl-2′-deoxycytidine (C5-methyl-dC) and 2′-deoxyinosine (dI) from Tokyo Chemical Industry Co., Ltd. (TCI, Tokyo, Japan); and N6-methyl-2′-deoxyadenosine (N6-methyl-dA) from Carbosynth Limited (Compton, Berkshire, UK). C5-hydroxymethyl-2′-deoxycytidine (C5-hydroxymethyl-dC) and N6-hydroxymethyl-2′-deoxyadenosine (N6-hydroxymethyl-dA) were synthesized according to previously reported protocols [24, 25].

### DNA adduct identification and semiquantification by LC-MS

LC-MS/MS analyses were performed using a 4000 QTRAP mass spectrometer (SCIEX, Framingham, MA, USA) with an Acquity UPLC system (Waters, Milford, MA, USA). Digested DNA (5 μl) was separated using an Acquity UPLC HSS T3 column (2.1 mm × 100 mm, Waters) with mobile phase A: 0.02% (v/v) acetic acid in water and mobile phase B: methanol at 0.2 mL/min in an isocratic and linear gradient elution program (3% B from 0 to 5 min; 3–15% B from 5 to 10 min; 15–80% B from 10 to 25 min; 80% B from 25 to 30 min). The target column temperature was 40 °C. Based on a previous report [17], selected reaction monitoring of 174 mass transitions was performed in positive ion mode using nitrogen as the nebulizing gas. The experimental conditions were as follows: ion source temperature, 400 °C; ion spray voltage, 5500 V; declustering potential, 51 V; entrance potential, 15 V; collision energy, 18 eV; and collision cell exit potential, 6 V.

### Data extraction from the chromatogram of each sample

A total of 59,334 chromatograms containing 341 samples for 174 mass transitions were imported into Skyline software [26]. After visual evaluation, 7 DNA adducts were selected because these analytes showed large peak areas, column retention times consistent with those of standard compounds and high prevalence in these tissue DNA samples. The mass transition values of these 7 DNA adducts are shown in Supplementary Table S1. For determination of each peak boundary, we first set a broad window covering the column retention time point of each standard compound (Supplementary Table S2). After confirming the highest peak in this broad window for each sample, a smaller window around the highest peak was defined as the “best retention time” in each chromatogram. Another window range of the same size outside the best retention time was defined as the “background”. No peak was present in these ranges in a blank sample (dH_2_O). The areas were calculated using these peak boundaries as measured in count per seconds (cps) x seconds (sec). Peak areas less than 500 were conservatively adjusted to 500. Peak areas with signal-to-noise ratios (best retention time/background) less than 3 were transformed to 0. To normalize signal drift between samples, a dilution factor was estimated using 6 mass transitions corresponding to intact deoxyribonucleosides or their isotopomers (precursor masses of 229.082, 229.094, 243.098, 244.093, 269.089 and 270.120). Peak areas of the 6 mass transitions were divided by the median value of each mass transition in samples for normalization. We used the median of the normalized peak area in 6 mass transitions generated in isotopomer measurements as the dilution factor for the samples and further used these values for normalization of the peak areas of 7 DNA adducts. The molar ratio of the DNA adduct to intact deoxyribonucleoside was calculated using data of standard compounds and their exact mass on the assumption that the GC content of human genomic DNA is 41%.

### Statistical comparison

For comparisons among individuals, we took the median of values from multiple sites of the stomach as the representative value for the individual. The Kruskal-Wallis test, Mann-Whitney U test, and Shapiro-Wilk test were conducted to evaluate interindividual and intraindividual differences and correlations with smoking and drinking. Spearman’s correlation analysis was used to calculate the correlation between age, body mass index (BMI), and 7 adducts. These analyses were performed using SPSS software version 26 (IBM, Chicago, IL) and Microsoft Excel 2016.

## Results

### Clinical profile

In 59 resected stomachs, samples were taken from up to 27 sites (mode = 4) per stomach (Fig. 1b and Supplementary Table S3). The summary and details of the basic profile, including age, sex and type of surgery, of the gastric cancer patients are described in Table 1 and Supplementary Table S4. The age ranged from 47 to 82 years old, and the mean (SD) was 68 (7.5). The mean (SD) of the BMI was 20 kg/m^2^ (3.9). The patients included 46 men and 13 women. All cases were clinically operable, and the stages ranged from I to III (there were no stage IV cases). Tumors were histologically classified according to JCS used in daily practice with 47 intestinal (tub1, tub2, por1) and 12 diffuse (sig, por2) histological types. Operative procedures included total resection in 27% and distal resection in 66%. The location, pathological details, and pTNM categories of the tumors are shown in Table 1. Lymph node dissection was performed in all cases, and 30 cases had lymph node metastases (N0: 29, N1: 10, N2: 8, N3: 12 total 59). The summary of the clinicopathological profile of the nongastric cancer cases (autopsy) was also added next to the resected cases in Table 1 and Supplementary Table S4. Regarding lifestyle information of gastric cancer cases, 24% were current smokers, 41% were never drinkers, and 17% were heavy drinkers. In autopsy cases, 6 out of 7 cases were ever smokers, and 4 out of 7 cases were current smokers. Five out of 7 were never drinkers, and 2 were heavy drinkers.

**Table 1:** Summary of subject clinicopathological information

	GC (*N* = 59)	Non-GC (*N* = 7)
	Mean (SD)	Mean (SD)
Age	68 (7.5)	72 (8.4)
BMI	20 (3.9)	19 (1.9)
	N (%)	N (%)
Sex		
Female	13 (22)	1 (14)
Male	46 (78)	6 (86)
Tobacco smoking		
Never	20 (34)	1 (14)
Former	25 (42)	2 (29)
Current	14 (24)	4 (57)
Alcohol consumption		
Never	24 (41)	5 (71)
Social drinking	12 (20)	0 (0)
Light	4 (7)	0 (0)
Moderate	9 (15)	0 (0)
Heavy	10 (17)	2 (29)
Operative procedure		
Total resection	16 (27)	
Proximal resection	2 (3)	
Distal resection	39 (66)	
Other (partial)	2 (3)	
T category		
T1a	12 (20)	
T1b	14 (24)	
T2	4 (7)	
T3	19 (32)	
T4a	9 (15)	
T4b	1 (2)	
N category		
N0	29 (49)	
N1	10 (17)	
N2	8 (14)	
N3a	8 (14)	
N3b	4 (7)	
Pathological stage		
IA	21 (36)	
IB	5 (8)	
IIA	7 (12)	
IIB	8 (14)	
IIIA	5 (8)	
IIIB	8 (14)	
IIIC	5 (8)	
Tumor location		
Upper	9 (15)	
Middle	26 (44)	
Lower	23 (39)	
Others	1 (2)	
Classification of cases by sampled gastric zone		
UML	9 (15)	
UM	4 (7)	
ML	42 (71)	
M	3 (5)	
Unknown	1 (2)	

The basic characteristics of gastric cancer (GC) or nongastric cancer (non-GC) subjects are summarized. Tumor-related information is also presented for GC cases. The details of this information are described in Supplementary Table S4

### DNA adducts identified in human stomach by LC-MS/MS

**Fig. 2:**
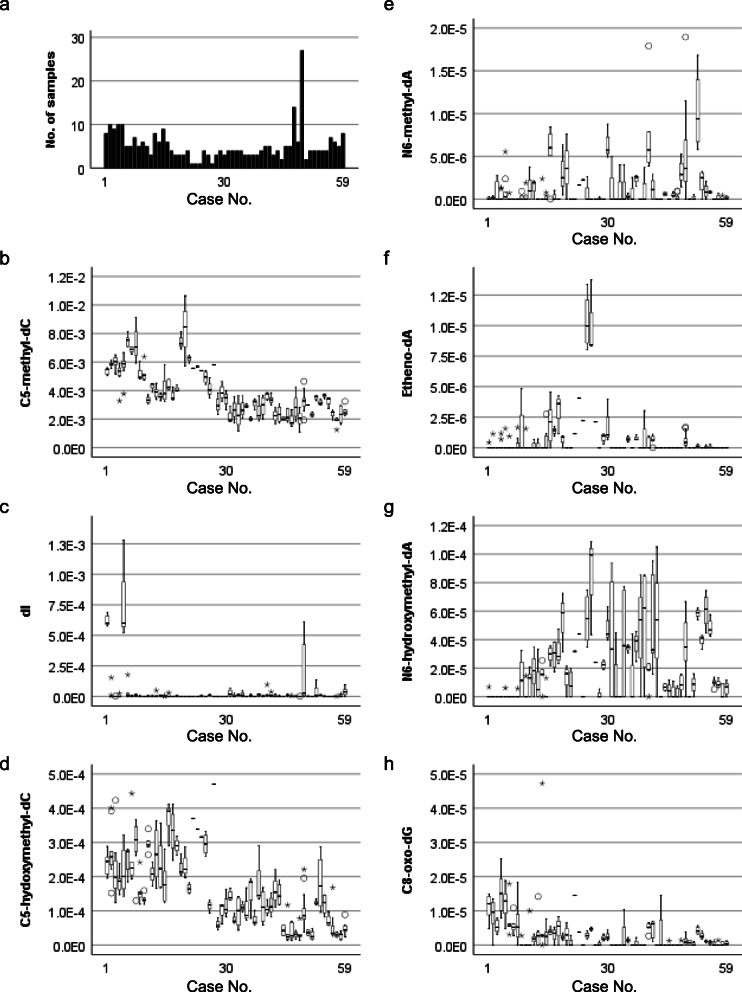
Interindividual variation in DNA adducts in 59 subjects, 306 samples, 1–27 per individual. (a) Number of samples in each case in which the measurements of the seven adducts were performed. (b-h) Box plots showing the molar ratio of DNA adducts in individuals (b: C5-methyl-dC, c: dI, d: C5-hydroxymethyl-dC, e: N6-methyl-dA, f: etheno-dA, g: N6-hydroxymethyl-dA, h: C8-oxo-dG). Values are shown in Supplementary Table S2. Zero values were included in the box plot calculation

**Fig. 3:**
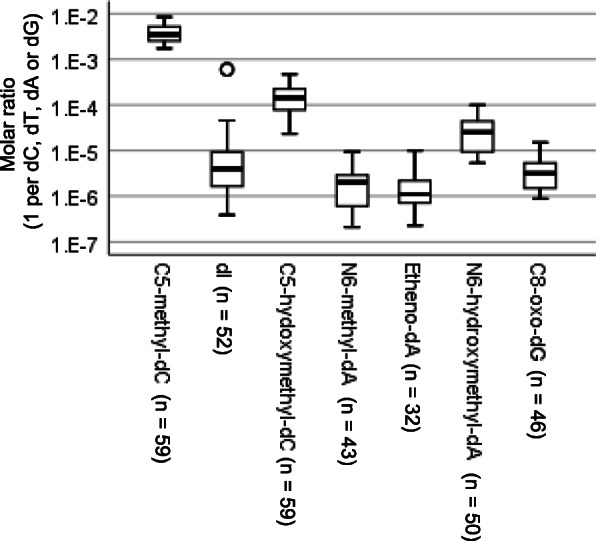
Distribution of DNA adduct quantities among individuals. The individual levels of DNA adducts in nontumor sites of gastric cancer cases are shown as box plots with a base-10 logarithmic scale. The center line of the box indicates the median, and the top and bottom edges of the box indicate their interquartile ranges (IQR). The whiskers show the maximum or minimum within a range of 1.5 times the IQR from the edge of the box. Outliers are indicated by circles (°) that are 1.5 to 3 times the IQR from the top or the bottom of a box and by stars (*) that are more than 3 times the IQR from the top or the bottom of a box. Zero values were excluded from the box plots

Each digested and electrospray-ionized nucleoside shows chromatographic peaks with defined m/z and column retention time. We picked up the peaks that were not observed in the blank sample and showed consistent column retention time and mass transition compared to standard compounds. Among 174 mass transitions, seven DNA adducts, including modified bases, C5-methyl-dC, dI, C5-hydroxymethyl-dC, N6-methyl-dA, etheno-dA, N6-hydroxymethyl-dA and C8-oxo-dG, were verified in human gastric mucosae. Representative chromatograms of standard compounds of 7 DNA adducts are shown in Supplementary Figure S1a-g. Semiquantitative distributions of each adduct are shown in Supplementary Figure S2. The interindividual range of the molar ratio of each adduct was larger than the intraindividual range (Fig. 2 and Supplementary Table S5, Kruskal-Wallis test, *p* = 0.00). As a representative value, we selected the median molar ratio in an individual. Figure 3 shows the distribution of the molar ratio of DNA adducts among individuals. As far as seven DNA adducts were characterized in our cohort, the distribution patterns did not fit a normal distribution (Supplementary Table S6, Shapiro-Wilk test, *p* = 0.00–0.01).

### The association of DNA adducts with clinical information

**Fig. 4:**
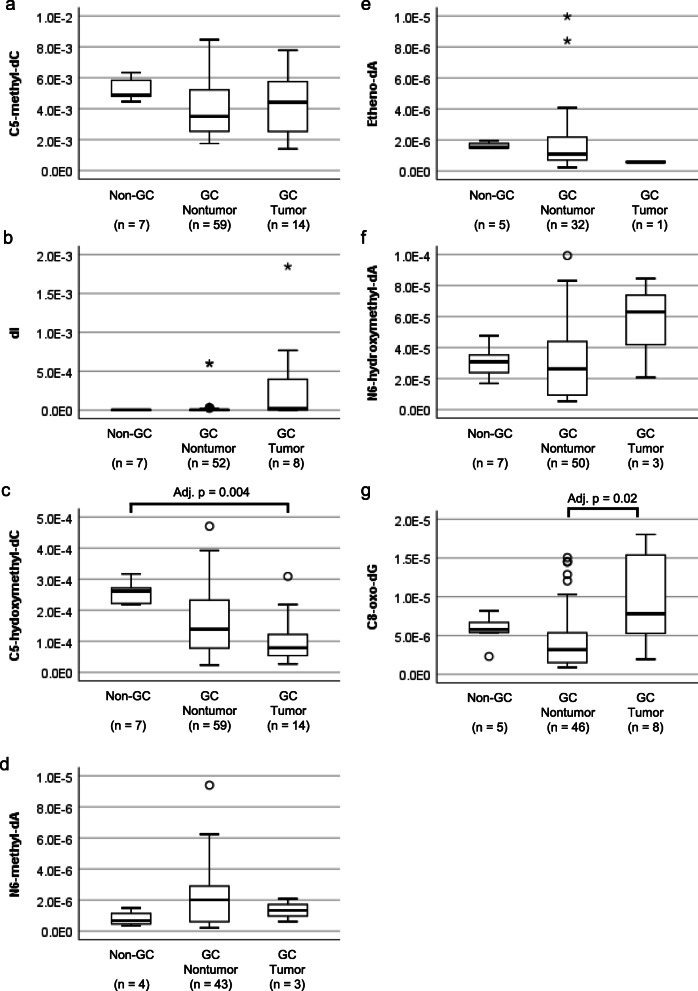
Quantities of DNA adducts in tumor and nontumor sites. Box plots of the molar ratio of DNA adducts in tumor or nontumor sites in gastric cancer subjects (GC tumor and GC nontumor) or in the stomachs of nongastric cancer subjects (non-GC) (a: C5-methyl-dC, b: dI, c: C5-hydroxymethyl-dC, d: N6-methyl-dA, e: etheno-dA, f: N6-hydroxymethyl-dA, g: C8-oxo-dG). The center line of the box indicates the median, and the top and bottom edges of the box indicate IQR. The whiskers show the maximum or minimum within a range of 1.5 times the IQR from the edge of the box. Outliers are indicated by circles (°) that are 1.5 to 3 times the IQR from the top or the bottom of a box and by stars (*) that are more than 3 times the IQR from the top or the bottom of a box. Kruskal-Wallis tests for the three groups and Bonferroni-corrected Mann-Whitney U tests for pairwise comparisons of the three groups confirmed the significant differences in 2 DNA adducts with adjusted *p*-values (adj. p) less than 0.05 (c, g). Zero values were excluded from the box plots

**Fig. 5:**
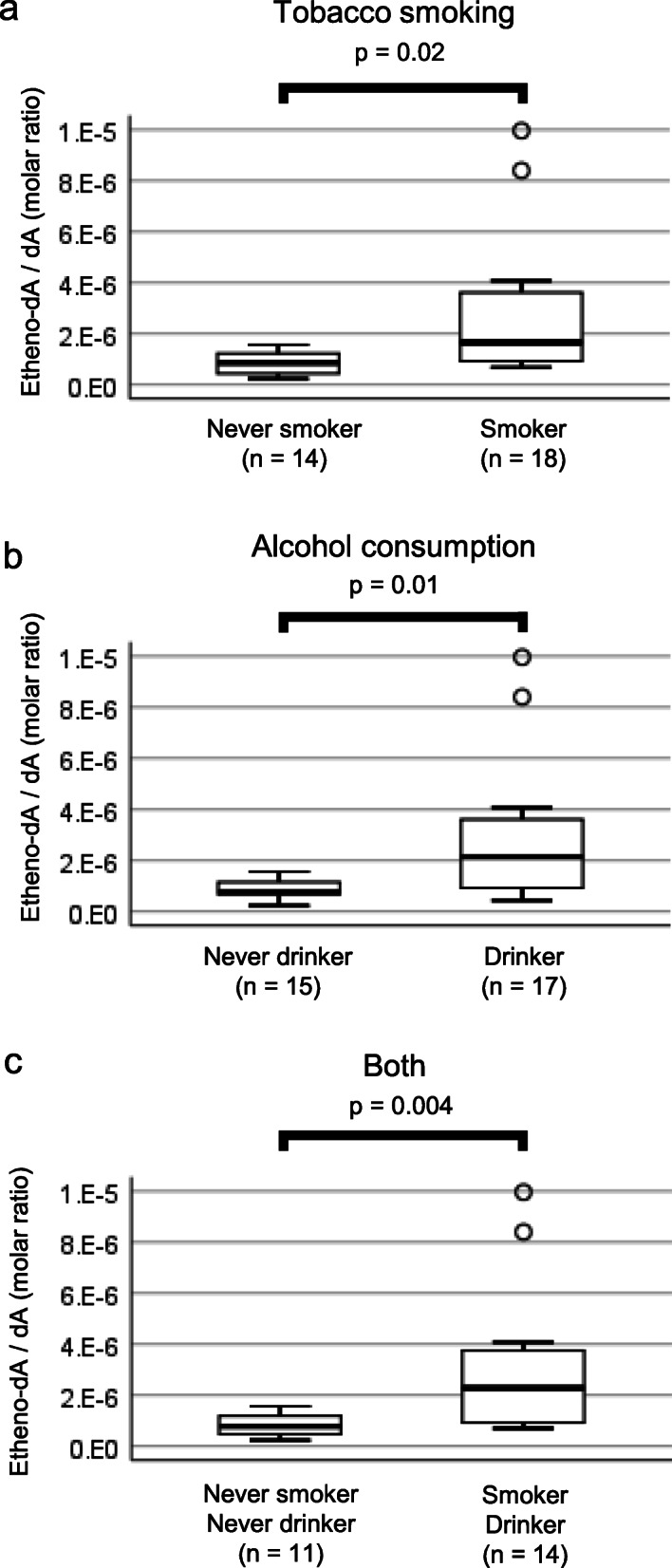
Association of DNA adduct levels with tobacco smoking and alcohol consumption. Box plots of etheno-dA show nominal p-values less than 0.05 by the Mann-Whitney U test in comparisons of (**a**) tobacco smoking, (**b**) alcohol consumption or (**c**) both. The center line of the box indicates the median, and the top and bottom edges of the box indicate their IQR. The whiskers show the maximum or minimum within a range of 1.5 times the IQR from the edge of the box. Outliers are indicated by circles (°) that are 1.5 to 3 times the IQR from the top or the bottom of a box and by stars (*) that are more than 3 times the IQR from the top or the bottom of a box. Zero values were excluded from the box plots

Figure 4 and Supplementary Table S7 show the comparison of DNA adducts in GC tumors (gastric cancer tumor portion), GC nontumors (gastric cancer nontumor portion), and non-GC (nongastric cancer) stomachs. C5-hydroxymethyl-dC was lower in GC tumors (adjusted *p* = 0.004) and GC nontumors (adjusted *p* = 0.05) than in non-GC tumors (Fig. 4c). C8-oxo-dG was higher in the tumor portion than in the corresponding nontumor portion (adjusted *p =* 0.02) (Fig. 4g). In terms of the correlation of adduct levels with lifestyle information, Supplementary Table S8 shows the difference in each adduct in never smokers vs smokers and never drinkers vs drinkers. Etheno-dA was greater in tobacco smokers than in never smokers, and it was also higher in drinkers than in never drinkers (Fig. 5a and b). The difference was clear in the comparison of the population who both smoked and drank with the population of never smokers and never drinkers (Fig. 5c). Correlations among 7 DNA adducts are shown in Supplementary Table S9. C5-methyl-dC was correlated with C5-hydroxymethyl-dC and C8-oxo-dG. dI was correlated with C8-oxo-dG. C5-hydroxymethyl-dC was further correlated with etheno-dA and C8-oxo-dG. N6-methyl-dA was correlated with N6-hydroxymethyl-dA. C5-hydroxymethyl-dC and C8-oxo-dG were the most frequent partners of the other adducts. No adducts investigated here had a high correlation of amount with age or BMI (Supplementary Table S9). Male subjects did not show a large difference in DNA adducts in the stomach compared with female subjects (Supplementary Table S8).

## Discussion

The demonstration of DNA adducts of any type is scarce, especially those using the LC/MS/MS approach. Matsuda et al. reported lipid peroxidation-derived DNA adducts in various human organs and verified the potential of the DNA adductome approach in human tissues [27]. Regarding the human gastric mucosa, we also showed the profiles of several lipid peroxidation-derived DNA adducts in gastric mucosa in gastric cancer patients from Japan and China [28]. In this research, we provided an overview of the 7 DNA adducts in multiple sites in a single stomach for the first time. C5-methyl-dC is a molecular entity of DNA methylation that is well known for its epigenetic regulation of gene expression [29]. C5-hydroxymethyl-dC is attracting attention as an oxidized form of C5-methyl-dC generated in the process of demethylation, or as a new epigenetic mark in itself. It has been argued whether N6-methyl-dA and N6-hydroxymethyl-dA also have epigenetic functions and whether a similar metabolic relationship holds, but it is not fully understood [25, 30]. Although dI is caused by deamination of deoxyadenosine, endonuclease V, which is a repair enzyme in bacteria, acts only on ribonucleoside in humans, and dI is considered to be potentially mutagenic [31]. Etheno-dA is one of the DNA adducts produced by lipid peroxide, and we first reported in a previous paper that it is present in the gastric mucosa [28]. C8-oxo-dG is one of the most studied DNA damages, caused by reactive oxygen species and associated with mutations [32].

The findings here showed that there were interindividual differences in the levels of these seven DNA adducts. As Fig. 3 shows, the level of etheno-dA ranged from undetectable to 10^− 5^ -10^− 6^ /dA, although the amount was semiquantitative. Many studies have assayed C8-oxo-2′-deoxyguanosine (also known as 8-OHdG or 8-oxoguanine) as a marker of reactive oxygen stress due to inflammation of gastric mucosa, but most of them used immunohistochemistry, ELISA systems, or polymer HRP detection systems using antibodies. Dyke et al. detected smoking-related DNA adducts in the human stomach [33]. In their report, they detected DNA adducts in gastric cancer tissues in 18 smokers and 8 nonsmokers by the ^32^P-postlabeling method. However, they did not identify the chemical nature of the spots on the autoradiographs, and they stated that “smoking-related” DNA adducts are more prevalent in smokers than in nonsmokers (in males than in females). Since the main method used to detect DNA adducts has changed from ^32^P-postlabeling to mass spectrometry [34], the series of findings from the detection and characterization of DNA adducts in human tissues are now expanding our understanding of the origin of the mutations occurring in human tissues.

In addition to DNA adducts generated by exogeneous chemicals, the base modifications enzymatically induced inside the body were characterized. Du et al. documented C5-formyl-deoxycytidine and C5-carboxy-deoxycytidine, both derived from C5-methyl-deoxycytidine, in matched pairs of gastric cancer and nontumor tissues and found barely detectable amounts of these two modified bases [35]. Interestingly, the amount of C5-hydroxymethyl-deoxycytidine was lower in the nontumor gastric mucosa of gastric cancer cases than in gastric mucosa without gastric cancer, which may reflect the general hypomethylation-hydroxylation process even in the adjacent field of carcinogenesis.

Our report here is the second to demonstrate the existence of etheno-dA in the stomach in Japanese individuals [28]. This lipid peroxidation-derived DNA adduct was more abundant in smokers and drinkers than in subjects who abstained from these habits. Age and sex, which have been supposed to influence the abundance of biomolecules, did not cause differences in the quantity of the 7 DNA adducts in the stomach. Therefore, it is reasonable to assume that there are other factors that explain the interindividual differences in at least 6 DNA adducts other than etheno-dA. Clinicopathological information that has not yet been considered, such as the degree of inflammation that can vary from individual to individual or among sampling sites, and genetic background, may be potential causes.

Xiao et al. observed N6-methyladenine DNA in matched human nontumor and tumor tissues of gastric and liver cancer [30] and noted a decrease in N6-methyladenine in tumors compared to the corresponding normal mucosa. In our cohort, the difference between tumor and nontumor tissue of the stomach was not prominent. We found N6-hydroxymethyl-dA in the human stomach for the first time. These DNA adducts were detectable in a limited population (N6-methyl-dA: 43/59, N6-hydroxymethyl-dA: 50/59) of our cohort above the threshold that was applied. Pathological, clinical, and biological investigation of these methylated bases is required to elucidate the environmental origin and significance of these adducts in the future.

The limitations of our study include that we characterized only a few of the adducts that we investigated. Modification of the DNA extraction, digestion, and purification protocols is under investigation for detecting various adducts, including higher molecular weights and more hydrophobic adducts. Finally, the quantification is still semiquantitative according to the limitations of the detection instrument.

## Conclusions

In summary, 7 DNA adducts and base modifications were detected in different amounts in multiple sites of the gastric mucosa. A reduction in C5-hydroxymethyl-dC even in the nontumor mucosa of patients with gastric cancer was observed. Smoking and drinking habits significantly influenced the quantity of one of the lipid peroxidation-derived adducts, etheno-dA. A more expansive DNA adductome profile with clinical and pathological details, in addition to characterization of the genetic changes of the tumors, would provide a comprehensive understanding of the origin of human cancer in the future.

## Supplementary Information

**Additional file 1.** Supplementary Figure S1: Chromatograms of standard compounds of DNA adducts. Representative chromatograms of blank (left panel) and standard compounds (right panel) in each mass transition and column retention times are shown (a: C5-methyl-dC, b: dI, c: C5-hydroxymethyl-dC, d: N6-methyl-dA, e: etheno-dA, f: N6-hydroxymethyl-dA, g: C8-oxo-dG). Supplementary Figure S2: Histogram of the quantity of each DNA adduct. The distributions according to the various amounts (molar ratios; horizontal axis) of DNA adducts (a: C5-methyl-dC, b: dI, c: C5-hydroxymethyl-dC, d: N6-methyl-dA, e: etheno-dA, f: N6-hydroxymethyl-dA, g: C8-oxo-dG) in nontumor sites of gastric cancer cases are shown. We assigned a zero value in some of the cases with considerably low or undetectable levels of adducts and depicted the histograms of molar ratios among 306 samples.

**Additional file 2 **Supplementary Table S1: Mass transitions of DNA adducts. Seven mass transitions that assume a neutral loss of 116 units of deoxyribose in DNA adducts of deoxyribonucleosides were monitored by mass spectrometry. Supplementary Table S2: Estimated molar ratios of DNA adducts in samples. As described in the Materials and Methods, the molar ratios of DNA adducts and three pairs of peak boundaries, namely, broad, best retention time (Best) and background (BG), were listed for each DNA adduct. A total of 341 samples were classified as GC (Case No. 1–59) and non-GC (Case No. 60–66). Samples from GC were also categorized as tumor or nontumor and are indicated in the Group Status column. Supplementary Table S3: Numbers of sampling sites. The number of samples per individual and its frequency are listed in the table of the nontumor portions of 59 GC cases. This is used to draw the histogram in Fig. 1b. Supplementary Table S4: Representative molar ratio of DNA adducts in individuals. Representative values of the molar ratio of DNA adducts in 59 cases of nontumor GC, 14 cases of tumor GC and 7 cases of non-GC were calculated. In addition to basic characteristics (age, sex, BMI, smoking, and alcohol), groups for comparison are indicated in Fig. 4 (Group Status) and Fig. 5 (Smoking Group, Alcohol Group and Smoking/Alcohol Group). To reduce redundancy, individual information for tumor samples was omitted, and the corresponding case number was indicated. Supplementary Table S5: Interindividual variance of DNA adducts. For the molar ratio of DNA adducts in 306 samples of nontumor gastric mucosae in 59 gastric cancer cases, the Kruskal-Wallis test was performed to compare individuals. Asymptotic significance calculated by SPSS software is displayed. The *p*-values less than 0.05 are marked in yellow. Supplementary Table S6: Normality test of the distribution of DNA adducts among individuals. The Shapiro-Wilk test was performed to determine the distribution of the representative molar ratio of DNA adducts in individuals. Cases with 0 values were excluded. The *p*-values less than 0.05 are marked in yellow. df: degrees of freedom. Supplementary Table S7: Accumulation of DNA adducts in tumor or nontumor sites of gastric mucosae. The Kruskal-Wallis test was performed to compare non-GC, GC nontumor and GC tumor samples. In the two DNA adducts showing *p*-values less than 0.05, Bonferroni-corrected pairwise Mann-Whitney U tests were also performed. The related box plots are drawn in Fig. 4. The p-values less than 0.05 are marked in yellow. Supplementary Table S8: Comparison of DNA adducts between sexes and among environmental exposure groups. The Mann-Whitney U test was performed to compare the molar ratio of DNA adducts in individuals with different characteristics, including sex, tobacco smoking, alcohol consumption and both habits. The data with p-values less than 0.05 are marked in yellow and are drawn as box plots in Fig. 5. Supplementary Table S9: Correlation analysis of age, BMI and 7 DNA adduct accumulations. Spearman’s rank correlation coefficient, rho and p-value were calculated pairwise among age, BMI and molar ratio of 7 DNA adducts. The p-values less than 0.05 are marked in yellow.

## Data Availability

All processed files are presented in additional files. Raw data files are available upon requests.

## Abbreviations

LC-MS/MS: Liquid chromatography and tandem mass spectrometry
U: Upper
M: Middle
L: Lower
JCS: the Japanese Classification System
IRB: Institutional Review Board
dC: 2′-deoxycytidine
dT: 2′-deoxythymidine
dG: 2′-deoxyguanosine
dA: 2′-deoxyadenosine
Etheno-dA: 1,N6-etheno-2′-deoxyadenosine
C8-oxo-dG: C8-oxo-2′-deoxyguanosine
C5-methyl-dC: C5-methyl-2′-deoxycytidine
dI: 2′-deoxyinosine
N6-methyl-dA: N6-methyl-2′-deoxyadenosine
C5-hydroxymethyl-dC: C5-hydroxymethyl-2′-deoxycytidine
N6-hydroxymethyl-dA: N6-hydroxymethyl-2′-deoxyadenosine
cps: count per seconds
BMI: Body mass index
GC: Gastric cancer
IQR: Interquartile ranges
