# Supplementary material for: Mass spectrometric profiling of DNA adducts in the human stomach associated with damage from environmental factors
Source: Genes Environ. 2021 Apr 9;43:12. doi: 10.1186/s41021-021-00186-2 (PMC8034090; doi:10.1186/s41021-021-00186-2)
Supplement: Supplementary file 1 — Additional file 1. Supplementary Figure S1: Chromatograms of standard compounds of DNA adducts. Representative chromatograms of blank (left panel) and standard compounds (right panel) in each mass transition and column retention times are shown (a: C5-methyl-dC, b: dI, c: C5-hydroxymethyl-dC, d: N6-methyl-dA, e: etheno-dA, f: N6-hydroxymethyl-dA, g: C8-oxo-dG). Supplementary Figure S2: Histogram of the quantity of each DNA adduct. The distributions according to the various amounts (molar ratios; horizontal axis) of DNA adducts (a: C5-methyl-dC, b: dI, c: C5-hydroxymethyl-dC, d: N6-methyl-dA, e: etheno-dA, f: N6-hydroxymethyl-dA, g: C8-oxo-dG) in nontumor sites of gastric cancer cases are shown. We assigned a zero value in some of the cases with considerably low or undetectable levels of adducts and depicted the histograms of molar ratios among 306 samples. [file 41021_2021_186_MOESM1_ESM.pptx]

## Slide 1
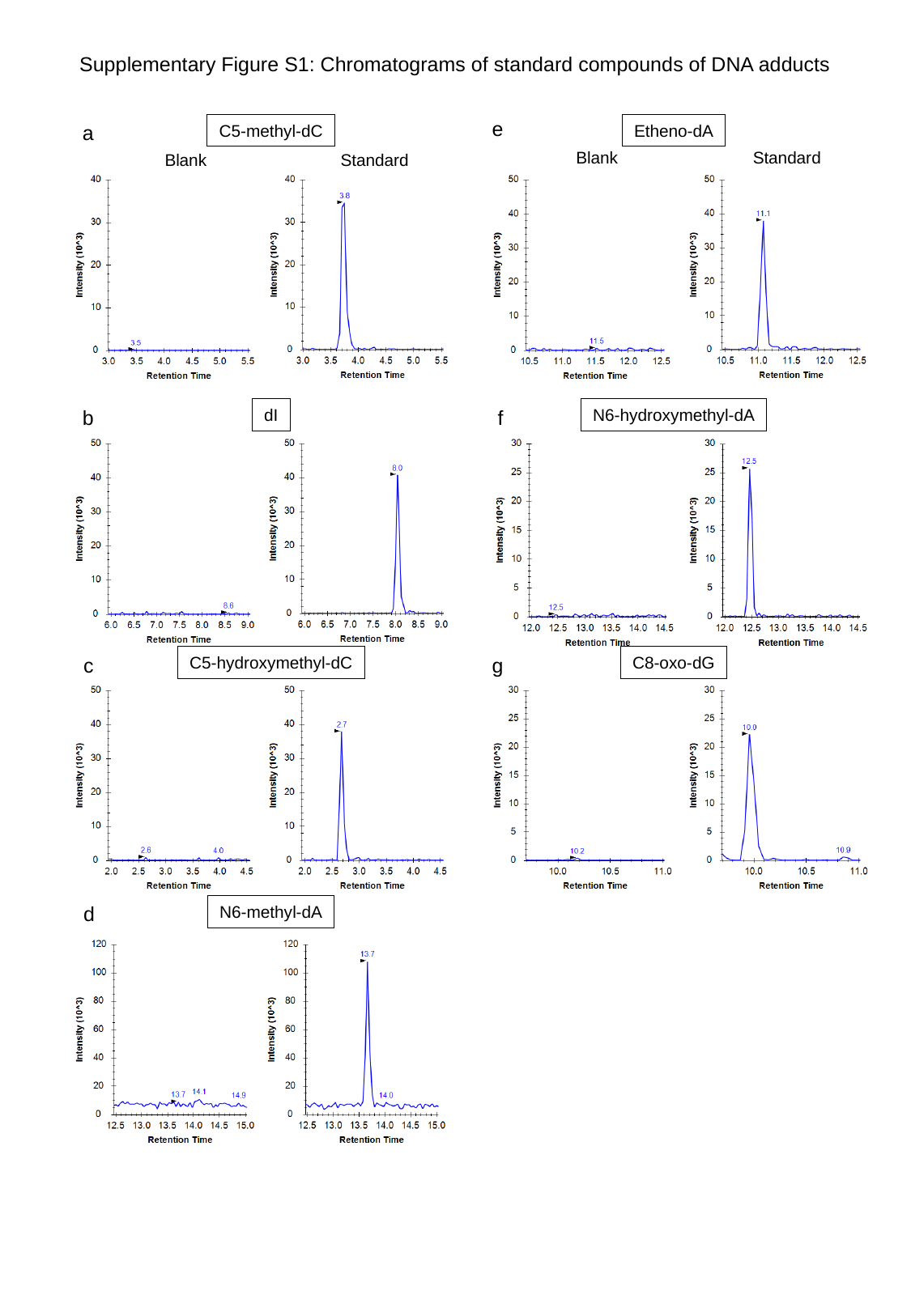

Supplementary Figure S1: Chromatograms of standard compounds of DNA adducts
e
a
C5-methyl-dC
Etheno-dA
Blank
Standard
Blank
Standard
b
dI
f
N6-hydroxymethyl-dA
c
C5-hydroxymethyl-dC
g
C8-oxo-dG
d
N6-methyl-dA

## Slide 2
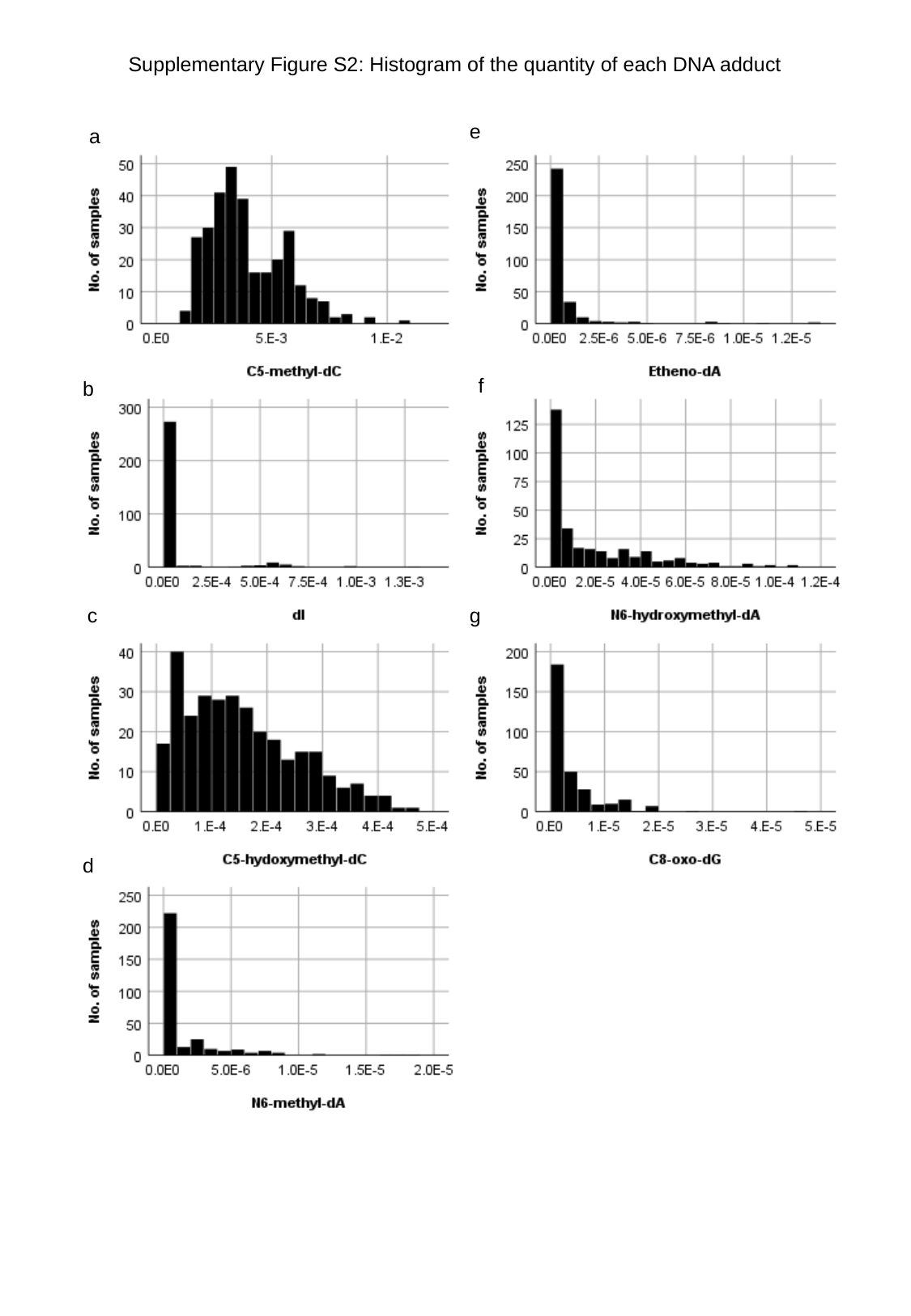

Supplementary Figure S2: Histogram of the quantity of each DNA adduct
e
a
f
b
c
g
d
